# Identification and Functional Analysis of Four RNA Silencing Suppressors in *Begomovirus* Croton Yellow Vein Mosaic Virus

**DOI:** 10.3389/fpls.2021.768800

**Published:** 2022-01-07

**Authors:** Ying Zhai, Anirban Roy, Hao Peng, Daniel L. Mullendore, Gurpreet Kaur, Bikash Mandal, Sunil Kumar Mukherjee, Hanu R. Pappu

**Affiliations:** ^1^Department of Plant Pathology, Washington State University, Pullman, WA, United States; ^2^Advanced Center for Plant Virology, Division of Plant Pathology, Indian Agricultural Research Institute, New Delhi, India; ^3^Franceschi Microscopy and Imaging Center, Washington State University, Pullman, WA, United States

**Keywords:** *Begomovirus*, **c**roton yellow vein mosaic virus, *Geminiviridae*, geminiviruses, protein-protein interaction, protein subcellular localization, silencing suppressor, virus movement

## Abstract

Croton yellow vein mosaic virus (CYVMV), a species in the genus *Begomovirus*, is a prolific monopartite begomovirus in the Indian sub-continent. CYVMV infects multiple crop plants to cause leaf curl disease. Plants have developed host RNA silencing mechanisms to defend the threat of viruses, including CYVMV. We characterized four RNA silencing suppressors, namely, V2, C2, and C4 encoded by CYVMV and betasatellite-encoded C1 protein (βC1) encoded by the cognate betasatellite, croton yellow vein betasatellite (CroYVMB). Their silencing suppressor functions were verified by the ability of restoring the β-glucuronidase (GUS) activity suppressed by RNA silencing. We showed here for the first time that V2 was capable of self-interacting, as well as interacting with the V1 protein, and could be translocalized to the plasmodesmata in the presence of CYVMV. The knockout of either V2 or V1 impaired the intercellular mobility of CYVMV, indicating their novel coordinated roles in the cell-to-cell movement of the virus. As pathogenicity determinants, each of V2, C2, and C4 could induce typical leaf curl symptoms in *Nicotiana benthamiana* plants even under transient expression. Interestingly, the transcripts and proteins of all four suppressors could be detected in the systemically infected leaves with no correlation to symptom induction. Overall, our work identifies four silencing suppressors encoded by CYVMV and its cognate betasatellite and reveals their subcellular localizations, interaction behavior, and roles in symptom induction and intercellular virus movement.

## Introduction

Viruses in the genus *Begomovirus* of the family *Geminiviridae* cause severe diseases in several crops and reduce their potential yield (Malathi et al., 2017). Begomoviruses possess circular single-stranded DNA genomes of either mono-(DNA-A) or bipartite (DNA-A and DNA-B) and are transmitted by whiteflies (*Bemisia tabaci*) (Seal et al., 2006). The genome of monopartite or each genomic component of the bipartite *Begomovirus* is 2.7–3.0 kb in size. In bipartite begomoviruses, DNA-A contains six partially overlapping open reading frames (ORFs) that encode the coat protein (CP, AV1) and precoat protein (AV2) in the viral sense strand, and the replication initiator protein (Rep, AC1), transcriptional activator protein (TrAP, AC2), replication enhancer protein (REn, AC3), and AC4 protein in the complementary strand. DNA-B contains two ORFs encoding nuclear shuttle protein (NSP, BV1) and movement protein (MP, BC1) in the viral-sense and complementary strands, respectively (Hanley-Bowdoin et al., 2013). Both DNA-A and DNA-B are required for systemic spread and symptom expression of bipartite begomoviruses with few exceptions (Stanley, 1983).

Some monopartite begomoviruses are virulent alone (Kheyr-Pour et al., 1991; Navot et al., 1991; Dry et al., 1993), but the majority of them require a satellite DNA molecule (betasatellite) to induce severe disease symptoms (Briddon et al., 2001; Jose and Usha, 2003; Cui et al., 2004; Saunders et al., 2004; Briddon and Stanley, 2006; Kon et al., 2009). Betasatellites are small, circular, single-stranded DNAs of about 1.3 kb in size and are often encapsidated within the viral particles. They depend on viral components for replication, encapsidation, and transmission. Only negligible sequence similarity can be found between betasatellites and DNA-A/DNA-B (Saunders et al., 2000; Briddon et al., 2003; Zhou et al., 2003), except a generally conserved sequence TAATATTAC, which forms a stem-loop structure in all begomoviruses. The betasatellite also contains an A-rich region and encodes the βC1 protein, which acts as a suppressor of gene silencing (Cui et al., 2005) and determines the host range of associated begomoviruses (Saunders et al., 2000, 2002; Jose and Usha, 2003).

Croton yellow vein mosaic virus (CYVMV), a species in the genus *Begomovirus*, is a prolific begomovirus in the Indian subcontinent. CYVMV was first reported from the weed host *Croton sparsiflorus* and later found on other crops such as *Jatropha gossypifolia* (Snehi et al., 2011), cluster bean (Zaffalon et al., 2012), radish (Singh et al., 2012), rapeseed-mustard (Roy et al., 2013), and Crambe (Kumar et al., 2018). Under the experimental conditions, a total of 35 plant species from 11 families have been reported as hosts of CYVMV (Pramesh et al., 2013). Although CYVMV infection causes bright yellow vein symptoms in its weed host (Pramesh et al., 2013), it produces the phenotype of the leaf curl disease in other crop plants or experimental hosts. As a monopartite begomovirus, CYVMV is often associated with a betasatellite named croton yellow vein betasatellite (CroYVMB) (Pramesh et al., 2013), which can also be transreplicated by other monopartite or bipartite begomoviruses and leads to an increased symptom severity (Swapna Geetanjali et al., 2013; Shilpi et al., 2015). Depending on host species, successful infection of CYVMV may or may not need the association of CroYVMB (Shilpi et al., 2015; Jailani et al., 2016), which indicates the versatility of CYVMV and the CroYVMB complex for the infection process.

Begomovirus infection triggers RNA silencing defense response in host plants (Teixeira et al., 2021). As a result of the plant-virus co-evolution, plant viruses encode RNA silencing suppressor proteins to counteract RNA silencing-based plant defense for efficient viral infection, replication, and systemic spread in host plants (Zhai et al., 2014). At least three begomovirus proteins, namely, AV2/V2, AC2/C2, and AC4/C4, have been shown to act as suppressors of RNA silencing (Bisaro, 2006) depending on the host species. Betasatellite-encoded C1 protein (βC1) can also exhibit a suppressor activity (Eini et al., 2012). These silencing suppressors often act as symptom determinants when expressed alone (Krake et al., 1998; Cui et al., 2005). The existence of multiple begomovirus silencing suppressors presents additional challenges for disease management. While C2 and βC1 suppress both transcriptional (TGS) and posttranscriptional gene silencing (PTGS) machineries of plants, V2 and C4 are found to specifically suppress PTGS. TGS and PTGS occur in the nucleus and cytoplasm of the plant cell, respectively. Both C2 and βC1 are found in the nucleus with βC1 of some species having additional chloroplast localization (Fondong, 2013). In contrast, both V2 and C4 are cytoplasmic proteins (Fondong, 2013). The subcellular localizations of these silencing suppressors are largely consistent with their respective TGS- or PTGS-suppressing roles. Begomovirus silencing suppressors interact with host proteins and modulate multiple pathways to exert their functions. However, it is not clear that whether they function independently or interact with each other to coordinate their activities.

In this report, we demonstrated that similar to other begomoviruses, CYVMV and its cognate betasatellite CroYVMB encode four silencing suppressors (i.e., V2, C2, C4, and βC1) to counteract RNA silencing-mediated plant defense mechanisms. To decipher the mechanism of suppressor functions, we determined the sub-cellular localization of the suppressors both in the presence or absence of the viral infection. We further analyzed the interactions of CYVMV silencing suppressors as well as their roles in pathogenicity and cell-to-cell movement.

## Materials and Methods

### Test of RNA Silencing Suppressor Activity

The CYVMV infectious clone has been described previously (Jailani et al., 2016). In the silencing suppressor activity test, wild-type *Nicotiana benthamiana* leaves and the *Agrobacterium* strain GV3101 were used for agroinfiltration assays as previously described (Zhai et al., 2021). Moderate GUS expression in *N. benthamiana* leaves was driven by the pG_N_/G_C_-CBSm promoter (Zhai et al., 2019). The GUS expression can be specifically silenced by the RNAi construct pRNAi-GG-GUS (Yan et al., 2012). The known virus-silencing suppressor HC-Pro was used as a positive control to suppress the GUS silencing effect of pRNAi-GG-GUS. Individual constructs expressing V2, C2, C4, or βC1 were co-agroinfiltrated to test their silencing suppressor activity. Empty vector (EV) was used as a negative control. Agroinfiltration combinations included pG_N_/G_C_-CBSm-GUS only, pG_N_/G_C_-CBSm-GUS + pRNAi-GG-GUS, pG_N_/ G_C_-CBSm-GUS + pRNAi-GG-GUS + HC-Pro, and pG_N_/G_C_-CBSm-GUS + pRNAi-GG-GUS + V2, C2, C4, or βC1. For vector construction, the coding sequences of V2, C2, C4, or βC1 were amplified by PCR using the Phusion proofreading DNA polymerase (Invitrogen, Carlsbad CA, United States) and virus-specific primers with attB adaptors. Each amplicon was first cloned into the pENTR/D-TOPO Gateway vector and then inserted into pEarleyGate103 (The Arabidopsis Information Resource, Arabidopsis Biological Resource Center) *via* the LR reaction. For agroinfiltration, *Agrobacterium* pellet from 50 ml culture was resuspended in the infiltration buffer (50 mM MES pH 5.6, 10 mM MgCl_2_, and 150 μM acetosyringone) and adjusted to an OD_600_ of 0.5. Four leaves from the same *N. benthamiana* plant were infiltrated with different combinations. For each combination, equal volumes of different cultures with same OD_600_ were mixed and used in agroinfiltration. Three biological replicates were taken for each experiment. All the primers used in this study are listed in Supplementary Table 1.

### β-Glucuronidase Staining and Quantification

β-Glucuronidase staining was performed as described previously (Jefferson et al., 1987). For the quantification of the GUS enzyme, fresh leaf samples inoculated with different constructs mentioned earlier were collected using a puncher and homogenized to fine powder by grinding in liquid nitrogen. For each sample, 200 μl GUS extraction buffer (50 mM NaHPO_4_, pH 7.0, 5 mM dithiothreitol, 10 mM Na_2_EDTA, 0.1% sodium lauryl sarcosine, and 0.1% Triton-100) was added, and 100 μl extract was mixed with 40 μl 10 mM fluorescent substrate of 4-methylumbelliferyl β-D-glucoside (MUG) (Sigma Life Science, St. Louis, MO, United States). After incubation at 37°C for 1 h, the reaction was stopped with 1 ml stop buffer (0.2 M sodium carbonate). After mixing well with the stop buffer, 100 μl of mixture was loaded onto a 96-well black microtiter plate (Greiner Bio-One, Monroe, NC, United States). Two replicate wells were carried out for each sample. The methylumbelliferone (MU) (Sigma Life Science, St. Louis, MO, United States) standard curve was included in each plate. Furthermore, 2.5 μM, 625 nM, 156 nM, and 39 nM MU gradients were used to generate the standard curve. The fluorescence intensity of MU was measured in a Molecular Devices SpectraMax M2 microplate reader (Molecular Devices Co., Sunnyvale, CA, United States).

### Protein Subcellular Localization

*V2*, *C2*, *C4*, and β*C1* genes were cloned into pEarleyGate103 with a C-terminal green fluorescent protein (GFP) fusion tag. These GFP fusion constructs were infiltrated individually or co-infiltrated with the CYVMV infectious clone into *N. benthamiana* leaves. After 3 days, infiltrated leaves were examined by a Leica confocal laser-scanning microscope to determine the subcellular localizations of the GFP fluorescent signal as described earlier (Sarkar et al., 2021). Leaves were incubated in 5 ng of Hoechst 33342 per ml of 1 × PBS buffer 20 min prior to imaging. A sequential scan was used to visualize the nucleic acid binding of Hoechst with excitation at 405 nm, and emission was collected from 420 to 470 nm; GFP fluorescence was visualized with an excitation of 488 nm and emission collected from 500 to 600 nm. Leaves were incubated in 1% aniline blue in tap water for 10 min to visualize plasmodesmata. A sequential scan was used with excitation of 405 nm and emission from 435 to 480 nm for aniline blue. The bright-field image to show cell walls was collected with a photomultiplier tube (PMT) detector, and GFP fluorescence of V2 CYVMV was imaged as above. In all GFP experiments, at least three individual plants inoculated with corresponding GFP constructs were used for confocal microscopy. For each plant, at least two leaves with at least three different areas from each leaf were chosen for the analysis. A representative image from each treatment was shown.

### Yeast Two-Hybrid Assay

Yeast two-hybrid (Y2H) assay was described previously (Peng and Neff, 2020). The coding sequences of V2, C2, C4, or βC1 were separately cloned into the Gateway compatible bait vector pBTM116-D9 and the prey vector pACT2-GW, respectively. The prey constructs were used to transform yeast strain A. For each event, a clone with the lowest self-activation was selected and transformed with the corresponding bait construct. The empty bait vector was used as a negative control. Positive protein-protein interactions were demonstrated by the ability of yeast clones to grow in the synthetic dropout IV (SDIV) media with uracil, histidine, leucine, and tryptophan being dropped. The known interaction between CIRCADIAN CLOCK ASSOCIATED 1 and ATAF2 (Peng and Neff, 2020) was used as a positive control. PCR-amplified sequences in all constructs used in this research were verified by sequencing.

### Bimolecular Fluorescence Complementation

The coding sequence of V2 was cloned into pSITE-cEYFP-N1 and pSITE-nEYFP-C1, respectively. Both constructs were then introduced into *Agrobacterium tumefaciens* GV3101 for *N. benthamiana* leaf infiltration. After a 48-h incubation, leaves were examined using a Leica TCS SP8 X confocal laser-scanning microscope, and the LAS X software (Leica Microsystems, Wetzlar, Germany) was used for image processing. The protein-protein interaction was indicated by the restoration of yellow fluorescent protein (YFP). YFP fluorescence was visualized with an excitation of 514 nm and emission collected from 530 to 600 nm. The bright-field signal to show the cell walls was collected with a PMT detector.

### Pull-Down Assay

The pull-down assay was performed as described previously (Peng and Neff, 2021). V1 and V2 proteins were fused with 6 × histidine (His) and maltose-binding protein (MBP) tags on their C- and N-terminals, respectively. Purified V1-His and MBP-V2 were mixed and passed through the amylose resin (New England Biolabs, Ipswich, MA, United States). After washing off unbound proteins, MBP-tagged proteins and their binding proteins were eluted for four times (i.e., E1 to E4) using elution buffer containing 10 mM maltose. As a negative control, purified V1-His and MBP tag protein were mixed and went through the identical process. Eluted proteins were separated by sodium dodecyl sulfate-polyacrylamide gel electrophoresis gel and stained with Coomassie brilliant blue R-250.

### V2 and V1 Knockout Constructs and Their Functional Assay

To create infectious knockout constructs, it is essential to create partial tandem dimeric repeat constructs where the gene to knockout is absent. To develop such constructs, initially, the intergenic region (IR) of the CYVMV was amplified from the genome of CYVMV using primers AR11 and AR12 and cloned into pGreenII vector using *Hin*dIII and *Eco*RI to create a pGreenII-IR construct. Similarly, a V2-IR region was amplified using AR11 and AR15 primers and cloned into the pGreenII vector using *Hin*dIII and *Eco*RI to create a pGreenII-V2-IR construct. These two initial constructs served the partial dimeric portion of the knockout constructs. Furthermore, a V2 deleted portion of CYVMV was amplified using primer combination AR13 and AR14 and cloned into pGreenII-IR using *Spe*I and *Not*I to generate V2-knockout construct of CYVMV (CYVMV-KoV2). Similarly, a V1 deleted portion of the CYVMV was amplified using primers AR17 and AR19 and cloned into pGreenII-V2-IR using *Spe*I and *Not*I to generate V1-knockout construct of CYVMV (CYVMV-KoV1). Next, a portion of the CYVMV genome containing the IR and C1, C2, and C3 ORFs was amplified using AR17 and AR14 and cloned into pGreenII-IR using *Spe*I and *Not*I to generate a double-knockout construct of V1 and V2 ORFs of CYVMV (CYVMV-KoV1-V2). All three knockout constructs were designed to retain the multiple cloning sites of *Eco*RI, *Xma*I, *Sma*I, and *Spe*I from pGreenII-IR. CYVMV-KoV1-GFP and CYVMV-KoV2-GFP constructs were generated by amplifying the *GFP* gene using AR20 and AR21 primers and cloning it into *Eco*RI/*Sma*I sites of the corresponding knockout constructs. The GFP expression was driven by the viral promoter in the IR region. Primer details are given in Supplementary Table 1. Primer locations and construct maps are illustrated in Supplementary Figure 1.

To understand the replication behavior of these constructs and the symptom phenotype produced by them, all the knockout constructs alone as well as with GFP were agroinfiltrated into *N. benthamiana* leaves. Inoculation of CYVMV agroinfectious clone served as a control. At 15 dpi, DNA was isolated from the agroinfiltrated tissue and systemically infected leaves. PCR was carried out using a set of abutting primers, i.e., AR40 and AR41, which were used to amplify the viral replicon. GFP epifluorescence was measured under UV light, and the intracellular GFP expression was analyzed using confocal microscopy. Maps of constructs and deletion mutants, episomal replicon released from the plasmid vector backbone, and locations of the abutting primers are shown in Figure 8.

### Pathogenicity Determination Assay

A set of five *N. benthamiana* plants were agroinfiltrated individually for each of the suppressor protein constructs in pEarleyGate103 vector; their impact on symptom development, if any, was monitored regularly. To assess the transportation of the transcripts of the suppressor gene constructs in the systemically infected leaf tissues, RNA was isolated from the systemically infected leaves of the suppressor construct-inoculated plants, and a reverse-transcriptase PCR (RT-PCR) was performed for pulled samples of three systemically infected leaves of all the five replications corresponding to all of the four suppressor-inoculated plants. The expression of the suppressor proteins in the systemically infected leaves was analyzed through confocal microscopy as described earlier.

## Results

### V2/C2/C4 and βC1 Are RNA-Silencing Suppressors From CYVMV and CroYVMB, Respectively

**FIGURE 1:**
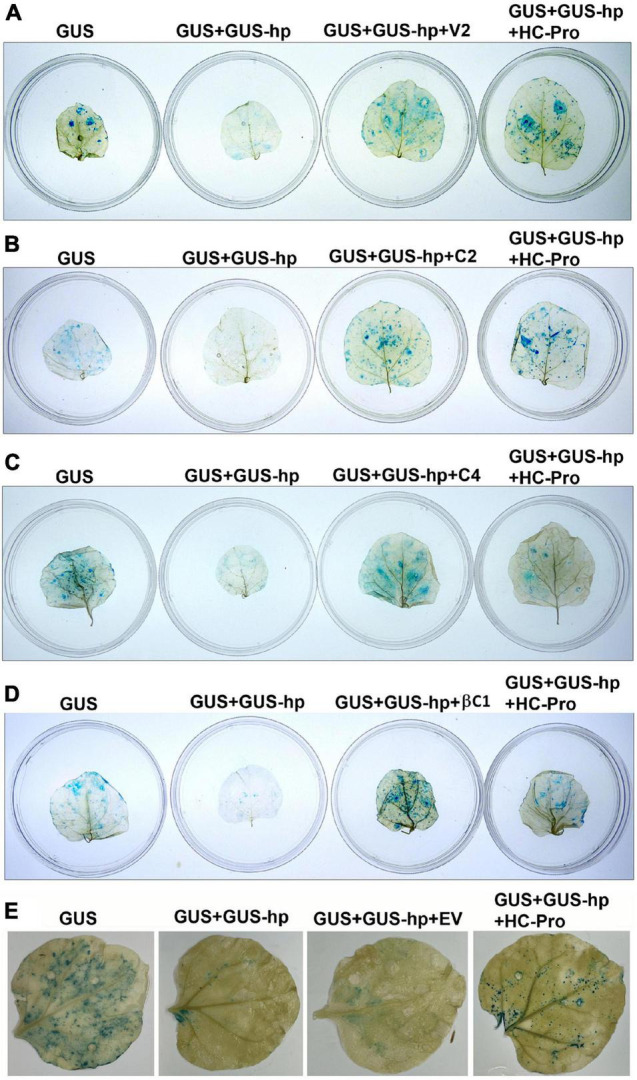
V2, C2, C4, and betasatellite-encoded C1 protein (βC1) are all RNA silencing suppressors from croton yellow vein mosaic virus (CYVMV) or croton yellow vein betasatellite (CroYVMB). pG_N_G_C_-CBSm:GUS (i.e., GUS) and pRNAi-GG-GUS (i.e., GUS-hp)-based GUS staining assays in agroinfiltrated *N. benthamiana* leaves demonstrated the silencing suppressor function of V2 **(A)**, C2 **(B)**, C4 **(C)**, and βC1 **(D)**. Co-infiltration of GUS-hp with GUS (GUS + GUS-hp) dramatically reduced GUS staining signals, indicating the suppression of GUS expression by RNA silencing. The third co-infiltrator of V2, C2, C4, or βC1 (GUS + GUS-hp + V2, GUS + GUS-hp + C2, GUS + GUS-hp + C4, or GUS + GUS-hp + βC1) led to the restoration of GUS signals, which demonstrate their silencing suppressor function. Potyvirus-encoded HC-Pro was used as a positive control (GUS + GUS-hp + HC-Pro). Co-infiltration of the empty vector (EV) pEarleyGate103 did not restore GUS signal suppressed by the RNAi construct GUS-hp **(E)**.

*Nicotiana benthamiana* plants showed the leaf curl symptom 10 days after co-agroinfiltration of a partial tandem repeat construct of CYVMV and a dimeric construct of CroYVMB. V2 (357 bp), C2 (405 bp), and C4 (258 bp) genes from CYVMV and βC1 (357 bp) gene from CroYVMB were amplified (excluding their stop codon) using the DNA isolated from these agroinfiltrated plants. Amplicons were cloned and sequenced to investigate the putative RNA silencing suppressor function of their encoded products. Transient silencing suppression assays were conducted on four leaves of the same *N. benthamiana* plant. After infiltration with *Agrobacterium* carrying pG_N_G_C_-CBSm:GUS (i.e., GUS), the leaves showed a moderate level of GUS expression. In contrast, dramatically reduced GUS staining signals were observed in leaves co-infiltrated with *Agrobacterium* carrying pG_N_G_C_-CBSm:GUS and pRNAi-GG-GUS (i.e., GUS-hp), indicating the suppression of the GUS expression by RNA silencing. When added with the third co-infiltrator of V2, C2, C4, or βC1 harbored by pEarleyGate103 (i.e., GUS + GUS-hp + V2, GUS + GUS-hp + C2, GUS + GUS-hp + C4, or GUS + GUS-hp + βC1), GUS signals were restored in all infiltrated leaves, demonstrating the silencing suppressor function of these four proteins (Figures 1A–D). The silencing suppressor HC-Pro (GUS + GUS-hp + HC-Pro) was used as a positive control for the restoration of the GUS signal. In the negative control assay, co-infiltration of the EV pEarleyGate103 did not restore GUS signal that was suppressed by GUS-hp (Figure 1E).

### GUS Quantification (MUG) Assays Reveal That V2 Can Restore GUS Activity Most Efficiently as Compared With the Other Three Suppressors

**FIGURE 2:**
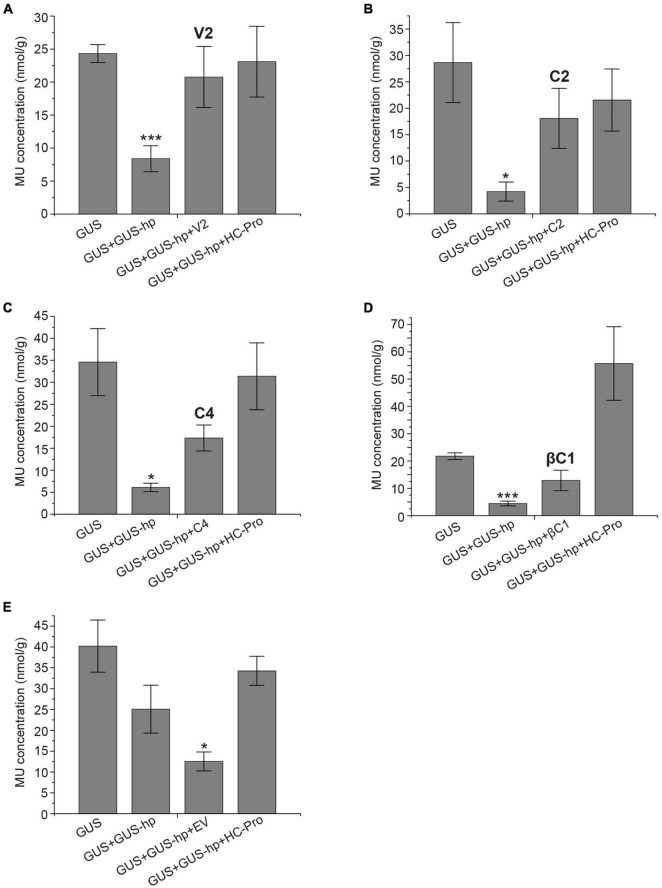
GUS quantification (MUG) assays confirm the silencing suppressor function of CYVMV V2 **(A)**, C2 **(B)**, C4 **(C)**, and βC1 **(D)**. HC-Pro was used as a positive control. EV was used as a negative control **(E)**. MU concentrations were presented as nmol per gram of leaf tissue (nmol/g). Each data point includes three biological replicates. Error bars denote standard error. Student’s *t*-test was used to determine the significance of differences. *P < 0.05 and ***P < 0.001.

Quantitative GUS expression assays were carried out to determine the silencing suppressor activity of V2, C2, C4, and βC1 (Figures 2A–D). All *N. benthamiana* leaves infiltrated with pG_N_G_C_-CBSm:GUS (i.e., GUS) showed similar levels of GUS accumulation, which was significantly reduced by the co-infiltration of pRNAi-GG-GUS (GUS + GUS-hp). Consistent with our qualitative results, additional co-infiltrations of V2, C2, C4 or βC1 (GUS + GUS-hp + Suppressor) all suppressed RNA silencing and thereby restored GUS accumulation, with V2 being the most effective silencing suppressor (∼85% GUS restoration efficiency). The restoration values with C2, C4, and βC1 were around 50–60%. As a positive control, HC-Pro consistently restored the GUS signal (Figures 2A–E). In contrast, EV exhibited no GUS restoration effect (Figure 2E).

### Subcellular Localization Assays Reveal That V2 Has an Altered Localization Behavior When Co-infiltrated With CYVMV

**FIGURE 3:**
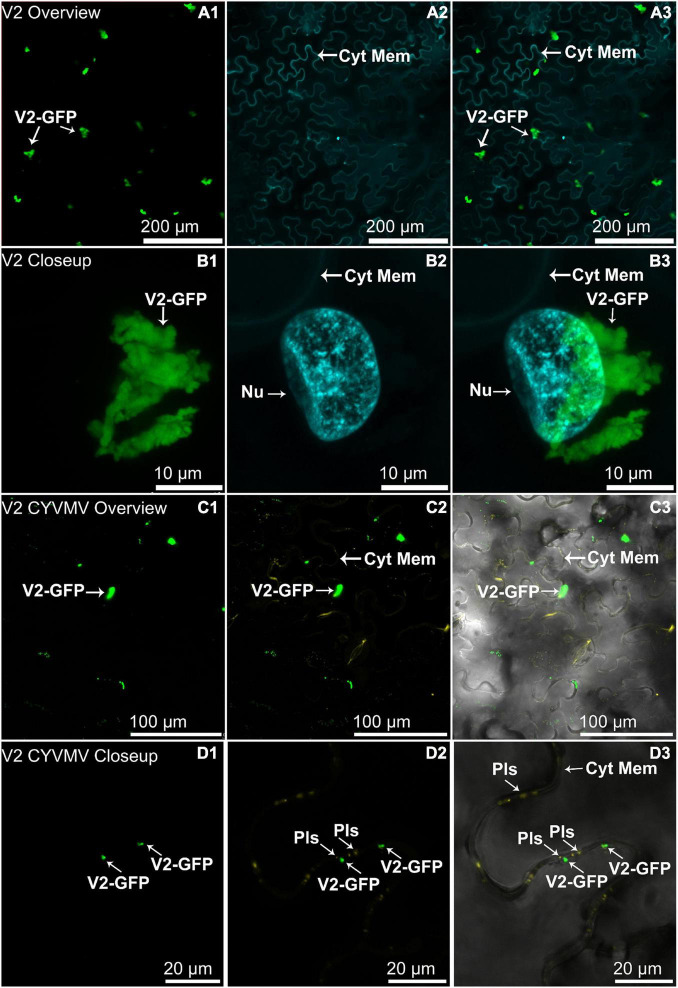
Subcellular localizations of enhanced green fluorescent protein (eGFP)-tagged V2 of CYVMV (CYVMV, V2-GFP) in *N. benthamiana* leaves under stand-alone **(A,B)** and CYVMV-inoculated **(C,D)** conditions. Panels **(A,C)** were taken under lower zoom, while panels **(B,D)** were taken with higher zoom. Images showing GFP fluorescence **(A1,B1,C1,D1)**, Hoechst fluorescence **(A2,B2)** and aniline blue bound to the plasmodesmata **(C2,D2)**. Merged images of both GFP and Hoechst fluorescence were shown as panels **(A3,B3)**. Merged GFP, aniline blue, and the bright field are shown in panels **(C3,D3)**. V2-GFP alone was found to aggregate as large clumps in various locations within the cytoplasm. Co-infiltration of V2-GFP and CYVMV turned some large V2-GFP clumps into smaller particles, which were loosely associated with the cell wall and moved to reach plasmodesmata. Arrowheads indicate the locations of V2-GFP and major sub-cellular organelle (Nu, nucleus; Pls, plasmodesmata; Cyt Mem, cytoplasmic membrane).

**FIGURE 4:**
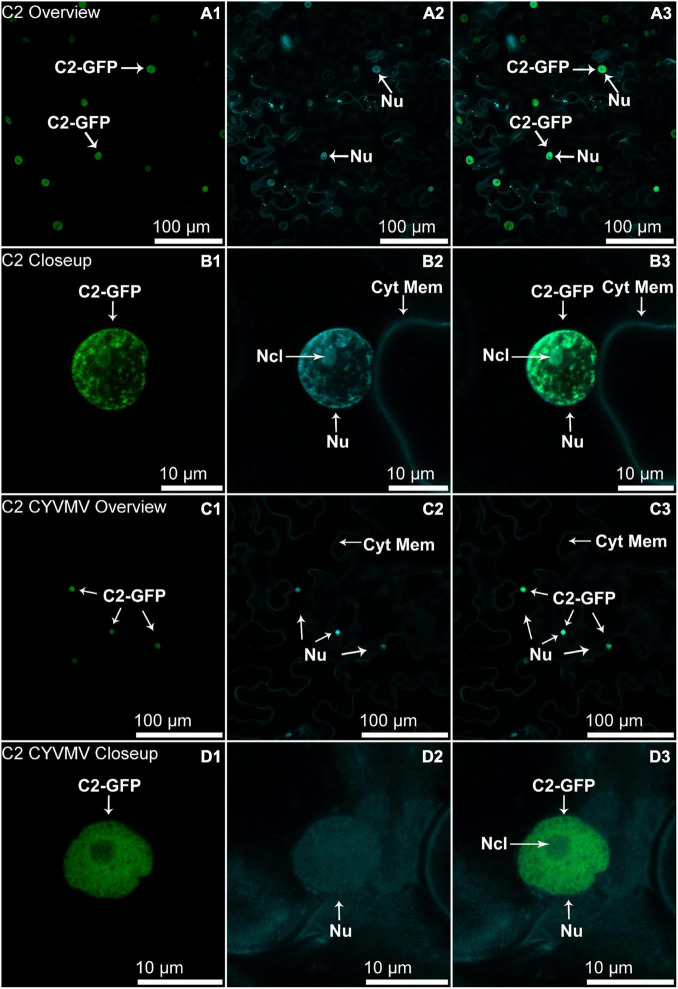
Subcellular localizations of CYVMV, C2-GFP in *N. benthamiana* leaves under stand-alone **(A,B)** and CYVMV-inoculated **(C,D)** conditions. Panels **(A,C)** were taken under lower zoom, while panels **(B,D)** were taken with higher zoom. Images showing GFP fluorescence **(A1,B1,C1,D1)**, and Hoechst fluorescence **(A2,B2,C2,D2)**. Merged images of both GFP and Hoechst fluorescence were shown as panels **(A3,B3, C3, D3)**. C2-GFP alone diffused in the nucleus, with most of the proteins located in the nucleoplasm. The presence of CYVMV did not dramatically change the subcellular localization pattern of C2-GFP except that its accumulation in the nucleolus was almost abolished. Arrowheads indicate the locations of C2-GFP and major sub-cellular organelle (Nu, nucleus, Ncl, nucleolus, Cyt Mem, cytoplasmic membrane).

**FIGURE 5:**
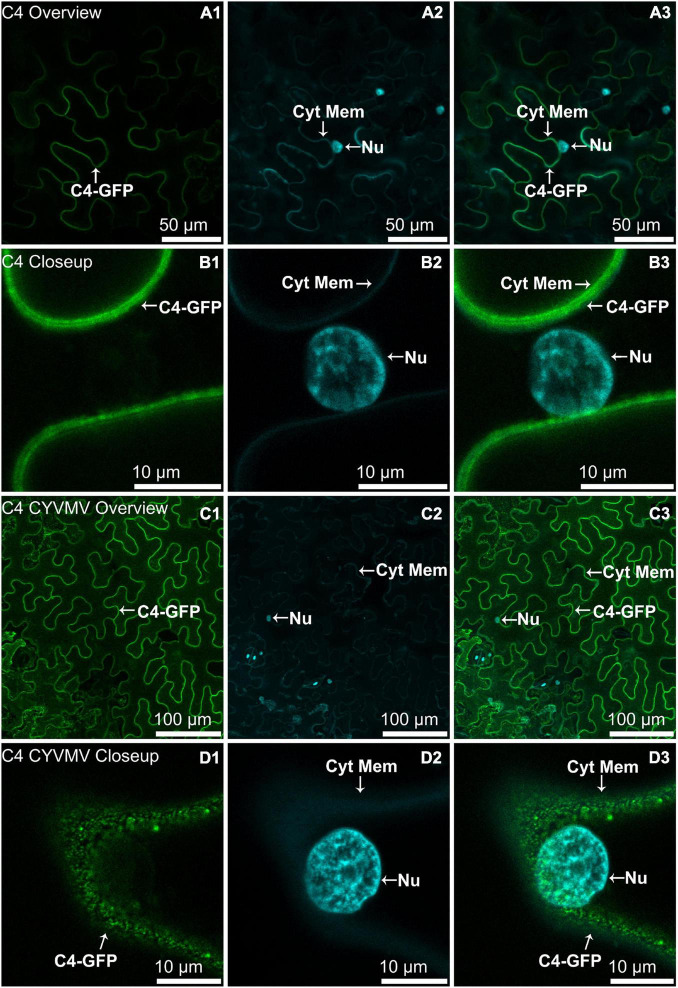
Subcellular localizations of CYVMV, C4-GFP in *N. benthamiana* leaves under stand-alone **(A,B)** and CYVMV-inoculated **(C,D)** conditions. Panels **(A,C)** were taken under lower zoom, while panels **(B,D)** were taken with higher zoom. Images showing GFP fluorescence **(A1,B1,C1,D1)** and Hoechst fluorescence **(A2,B2,C2,D2)**. Merged images of both GFP and Hoechst fluorescence were shown as panels **(A3,B3,C3,D3)**. The C4-GFP localization appeared to be consistently in the plasma membrane with or without CYVMV inoculation. CYVMV can induce the aggregation of C4-GFP into small particles within the membrane. Arrowheads indicate the locations of C4-GFP and major sub-cellular organelle (Nu: nucleus; Cyt Mem, cytoplasmic membrane).

**FIGURE 6:**
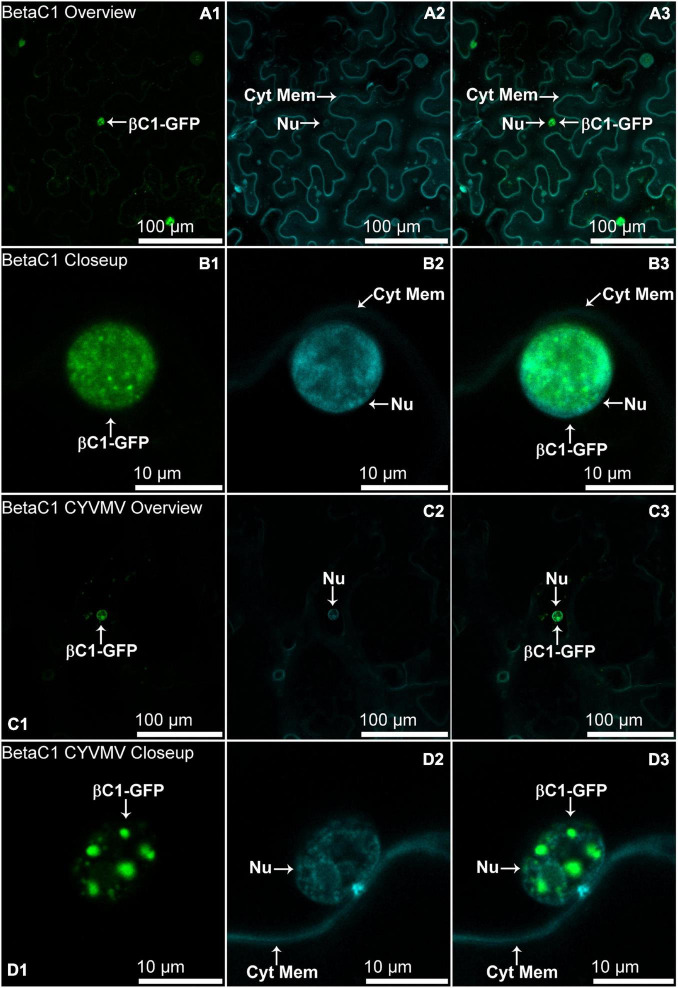
Subcellular localizations of CroYVMB, βC1-GFP in *N. benthamiana* leaves under stand-alone **(A,B)** and CYVMV-inoculated **(C,D)** conditions. Panels **(A,C)** were taken under lower zoom, while panels **(B,D)** were taken with higher zoom. Images showing GFP fluorescence **(A1,B1,C1,D1)**, and Hoechst fluorescence **(A2,B2,C2,D2)**. Merged images of both GFP and Hoechst fluorescence were shown as panels **(A3,B3,C3,D3)**. Both βC1-GFP and βC1-GFP + CYVMV showed strong GFP fluorescent signal in the nucleus. Arrowheads indicate the locations of βC1-GFP and major sub-cellular organelle (Nu: nucleus; Cyt Mem, cytoplasmic membrane).

The nucleus and cytoplasm localizations of silencing suppressors may indicate their suppression effect related to TGS and PTGS, respectively. Hence, the subcellular localizations of V2, C2, C4, and βC1 were examined using their GFP fusion proteins in the absence and presence of CYVMV. In our assays, enhanced green fluorescent protein (eGFP)-tagged V2 (V2-GFP) alone was found to aggregate as large clumps in various locations within the cytoplasm, mainly surrounding the nucleus (Figures 3A,B). Co-infiltration of V2-GFP and CYVMV showed a weaker GFP signal loosely associated with the cell wall and moved to reach the plasmodesmata (Figures 3C,D). C2-GFP alone diffused in the nucleus, with most of the proteins located in the nucleoplasm (Figures 4A,B). The presence of CYVMV did not dramatically change the subcellular localization pattern of C2-GFP except that its accumulation in the nucleolus was almost abolished (Figures 4C,D). Furthermore, the nucleus of C2-GFP and CYVMV co-infiltrated plants showed a slight dilation in size and deformation of spherical shape. The C4-GFP localization appeared to be consistently in the plasma membrane with or without CYVMV inoculation. CYVMV can induce the aggregation of C4-GFP into small particles within the membrane (Figure 5). Both βC1-GFP and βC1-GFP + CYVMV showed a strong GFP fluorescent signal in the nucleus (Figure 6). Overall, only V2 localization was affected by CYVMV. It is possible that V2 interacts with one or more other viral proteins or with host factors that are altered by infection and even with the CYVMV genome. Such interactions may facilitate V2 relocalization and subsequent cell-to-cell transfer *via* the plasmodesmata.

### V2 Physically Interacts With Itself and CYVMV V1

**FIGURE 7:**
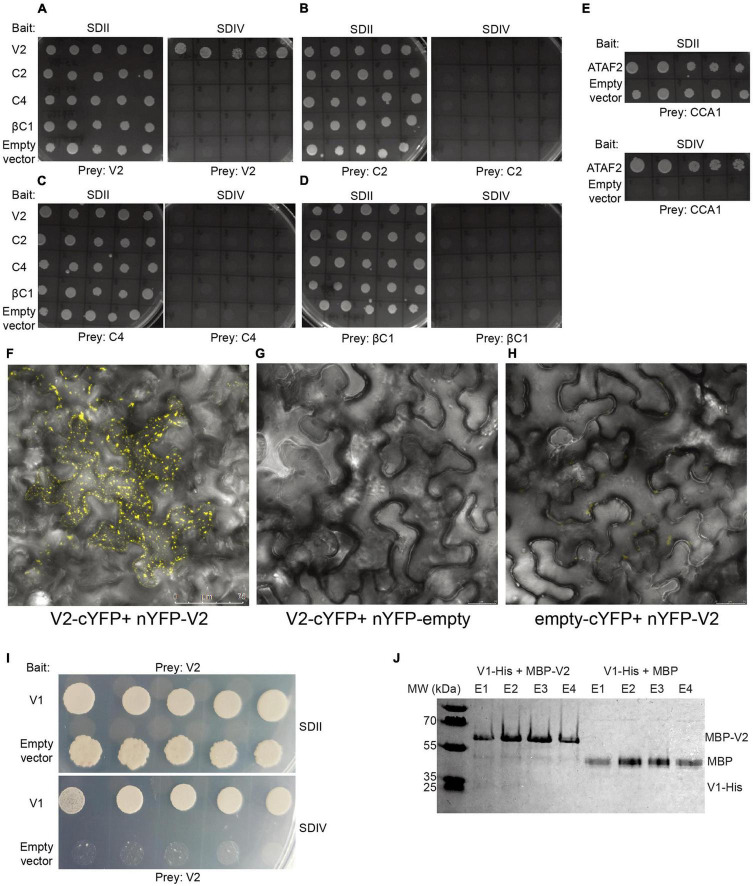
CYVMV V2 physically interacts with itself and V1. When testing the self and mutual interactions among all four silencing suppressors V2 **(A)**, C2 **(B)**, C4 **(C),** and βC1 **(D)** *via* targeted Y2H, only V2 exhibits self-interaction. Empty bait vector pBTM116-D9 was used as the negative control. A known interaction between *Arabidopsis* CCA1 and ATAF2 was used as a positive control **(E)**. All combinations can grow on SDII plates deprived of leucine and tryptophan, as the bait and prey vectors provide the biosynthesis ability of these two amino acids. Only the positive V2-V2 combination can grow on synthetic dropout IV (SDIV) plates, which indicates their physical interaction. The interaction increases the ability of uracil and histidine biosynthesis in yeast cells and thereby enables their growth on SDIV deprived of uracil, histidine, leucine, and tryptophan. Five independent yeast clones were shown for each combination. Self-interaction of V2 was further confirmed by the *in planta* bimolecular fluorescence complementation (BiFC) approach. **(F)** *N. benthamiana* leaves infiltrated with V2-cYFP + nYFP-V2 constructs showed strong yellow fluorescent signals. As negative controls, no fluorescence was detected in leaves infiltrated with V2-cYFP + nYFP-empty **(G)** or empty-cYFP + nYFP-V2 **(H)**. **(I)** CYVMV V1 and V2 proteins physically interact with each other in a targeted Y2H assay. Five independent yeast clones were shown. **(J)** V1-V2 interaction was confirmed in a pull-down assay. V1-His + MBP-V2 and V1-His + MBP protein mixtures were loaded to pass through the amylose resin, respectively. After washing away unbound proteins, four rounds of elution (E1-E4) were performed to collect maltose-binding proteins and their interactors. V1-His and MBP-V2 were co-eluted from the amylose resin (E1 and E2). In contrast, the MBP tag protein cannot bind V1-His. YFP, yellow fluorescent protein.

Whether the CYVMV silencing suppressors are capable of physically interacting with one another and themselves are yet to be investigated. Virus might use such interactions for their own replication and spread. Thus, potential self- or mutual interactions among V2, C2, C4, and βC1 were tested by targeted Y2H assays (Figures 7A–E). Only the self-interaction of V2 was observed in all combinations (Figure 7A). This result indicates that V2 may fulfill its silencing suppressor function in the form of a homodimers or multimers. C2, C4, and βC1 may suppress RNA silencing individually with no involvement of self-multimerization.

To further confirm the Y2H result, *in vivo* self-interaction of V2 was tested using bimolecular fluorescence complementation. The full-length V2 gene was cloned to fuse with N- and C-terminal fragments of YFP and co-infiltrated onto the *N. benthamiana* leaves. Infiltration with V2-cYFP + nYFP-V2 constructs resulted in strong yellow fluorescent signals (Figure 7F). As negative controls, no fluorescence was detected in leaves infiltrated with V2-cYFP + nYFP-empty (Figure 7G) or empty-cYFP + nYFP-V2 (Figure 7H). These results confirmed the self-interaction of V2 *in planta*.

Since subcellular localization results (Figure 3) suggest that V2 may interact with other viral protein(s) and involve in the CYVMV cell movement, its protein binding capacity was further investigated. In a targeted Y2H assay, CYVMV V1 and V2 proteins physically interact with each other (Figure 7I). The interaction was confirmed by a pull-down assay (Figure 7J). The physical interaction between V1-His and MBP-V2 ensured their co-elution from the amylose resin.

### Both V1 and V2 Are Required for CYVMV Movement

**FIGURE 8:**
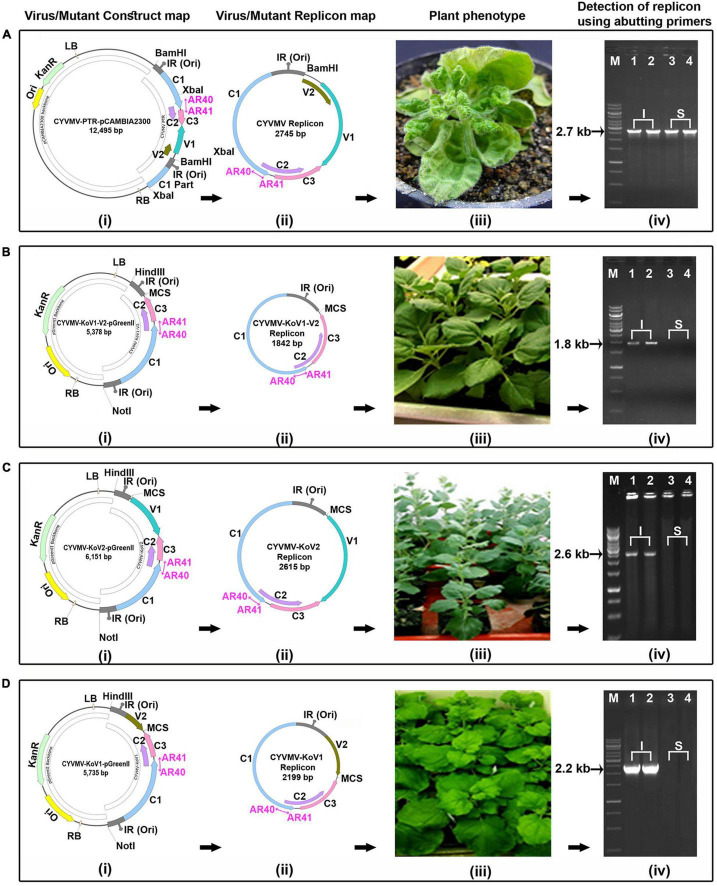
Both CYVMV V1 and V2 are required for CYVMV movement. Infiltrations of CYVMV infectious clone (CYVMV-PTR-pCAMBIA2300) **(A)** and deletion constructs of V1/V2 double (CYVMV-KoV1-V2-pGreenII) **(B)**, V2 single (CYVMV-KoV2-pGreenII) **(C)** and V1 single (CYVMV-KoV1-pGreenII) **(D)** to *N. benthamiana* leaves for the infectivity assay. Construct maps (i), viral/deletion episomal replicons released from vector backbone inside the plant (ii), symptom phenotype (iii), and detection of viral/mutant replicons using abutting primers (AR40, AR41) in infiltrated (I) and systemic (S) leaves (iv). All three deletion constructs did not cause any symptoms and were detected only in infiltrated leaves. In contrast, inoculation of the wild-type CYVMV led to infection symptoms, and the virus was present in both infiltrated and systemically infected leaves. Locations of the abutting primers AR40 and AR41 were marked as violate color arrows in the constructs and replicons.

**FIGURE 9:**
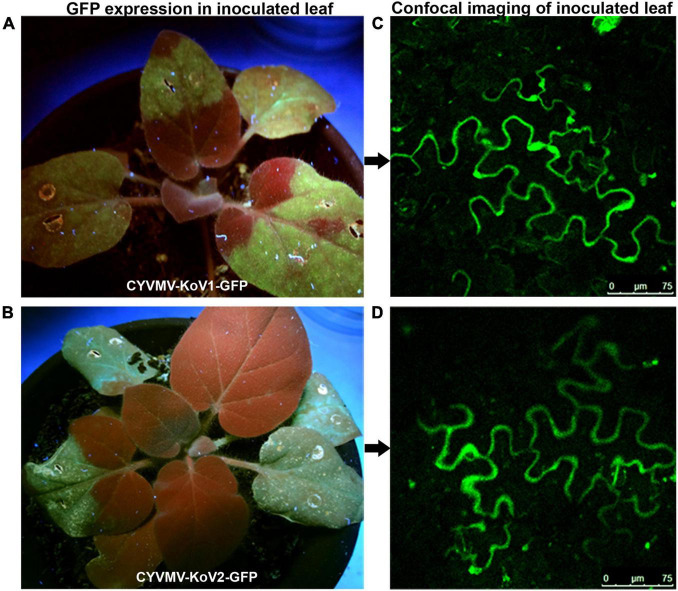
GFP-fused CYVMV V1 (CYVMV-KoV1-GFP) and V2 (CYVMV-KoV2-GFP) deletion constructs were created for individual *N. benthamiana* leaf infiltrations. GFP expression evidenced through epifluorescence **(A,B)** and confocal imaging **(C,D)**. Deletion of either V1 or V2 restricted GFP signals within the infiltration sites.

Since V1 and V2 interact, we investigated whether such an interaction is necessary for the cell-to-cell movement of the virus. V1/V2 single and double deletion (CYVMV-KoV1, CYVMV-KoV2, and CYVMV-KoV1-V2) constructs were created to infect *N. benthamiana* plants followed by molecular assays. All three deletion CYVMVs were detected only in infiltrated leaves (Figure 8) when using the abutting primers designed from C1 ORF. In contrast, wild-type CYVMV was present in both infiltrated and systemically infected leaves (Figure 8A). Abutting primers are a set of divergent primers whose 5′ end has two consecutive nucleotides in two strands. They specifically amplify the circular viral genome from the outward direction (Figure 8). We used such divergent primers to investigate whether the virus mini genomes (deletions of either V1 or V2 or both ORFs) can be released from the vector backbone and start replication as an episome. GFP-fused V1 (CYVMV-KoV1-GFP) and V2 (CYVMV-KoV2-GFP) deletion constructs were created for infiltrating individual *N. benthamiana* leaves. The GFP expression was monitored through epifluorescence and confocal imaging. Deletion of either V1 (Figures 9A,C) or V2 (Figures 9B,D) restricted GFP signals within the infiltration sites. Taken together, these results above demonstrate that the absence of either V1 or V2 inhibits the cell-to-cell CYVMV movement but not virus replication (Figures 8, 9).

V1 and V2 deletion constructs were complemented with co-inoculation of V2 and V1 constructs driven by the same right-side bidirectional promoter, respectively. Both complementation efforts failed to restore the movement function (data not shown). Since V1 and V2 ORFs were carried by two different constructs for complementation, the C2 protein might be incapable of activating the promoters of individual V1 and V2 ORFs to generate enough transcripts, which could lead to the complementation failure.

### V2, C2, and C4 Are Pathogenicity Determinants With Transcripts Moving Systemically

**FIGURE 10:**
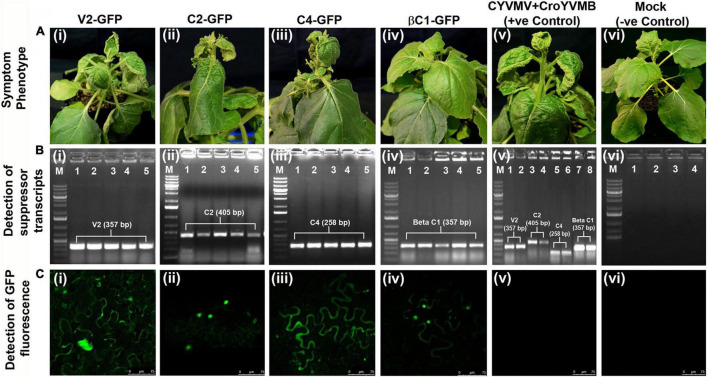
Pathogenicity determination assays of the four silencing suppressors from CYVMV or CroYVMB. Pathogenicity induced by V2, C2, C4, or βC1 was determined *via* agroinfiltrating their respective overexpression constructs (fused with eGFP in pEarleyGate103) to *N. benthamiana* leaves. Five plants were infiltrated for each construct. **(A)** Variable leaf curl symptoms can be detected in plants infiltrated with V2-GFP, C2-GFP, or C4-GFP constructs **(Ai–iii)** but not βC1-GFP-infiltrated plants **(Aiv)**. CYVMV and CroYVMB infiltrated (i.e., the positive control) plants also showed similar symptoms **(Av)**. As expected, no symptom was observed with mock infiltrated plants (i.e., the negative control) **(Avi)**. **(B)** Similar as the positive control, transcripts of all four silencing suppressor genes can be detected in systemically infected leaves of their respective infiltrated plants regardless of the symptomatic or asymptomatic phenotypes **(Bi–v)**. The negative control did not generate any amplification **(Bvi)**. **(C)** Consistently, confocal microscopy detected expression of the respective GFP-tagged suppressor proteins in all systemically infected leaves **(Ci–iv)**. As expected, GFP expression was not observed in either positive or negative controls **(Cv,vi)**.

Since silencing suppressors often act as symptom determinants when expressed alone, pathogenicity tests were performed for the four CYVMV silencing suppressors. Levels of pathogenicity induced by V2, C2, C4, or βC1 were determined *via* agroinfiltrating their respective overexpression constructs (fused with eGFP in pEarleyGate103) to *N. benthamiana* leaves. Five plants were infiltrated for each construct. Four, two, and three out of five infiltrated plants showed variable leaf curl symptoms with regard to V2-GFP, C2-GFP, and C4-GFP constructs, respectively (Table 1 and Figures 10Ai–iii). In contrast, none of the βC1-GFP-infiltrated plants exhibited any symptoms (Table 1 and Figure 10Aiv). All five plants infiltrated with CYVMV and CroYVMB constructs (i.e., positive control) also showed the similar leaf curl symptom (Figure 10Av), which was not observed in the mock infiltrated plants (i.e., negative control) (Table 1 and Figure 10Avi). Similar to the positive control, transcripts of all four silencing suppressor genes could be detected in systemically infected leaves of their respective infiltrated plants regardless of the symptomatic or asymptomatic phenotypes (Figures 10Bi–v). No amplification was obtained from the negative control (Figure 10Bvi). Consistently, the expression of the respective GFP-tagged suppressor proteins in all systemically infected leaves was found by confocal microscopy (Figures 10Ci–iv) on which no wild-type virus was inoculated. As expected, no GFP fluorescence was observed in either positive or negative controls (Figures 10Cv,vi).

**TABLE 1:** Pathogenicity determination of croton yellow vein mosaic virus (CYVMV)-encoded V2, C2, C4, and betasatellite-encoded C1 protein (βC1) through their transient overexpression in *N. benthamiana*.

Construct	Symptoms	Days needed for symptom appearance	Frequency of infection*
V2-GFP	Severe curling of leaves, enation, vein thickening, small leaves	12–15	4/5
C2-GFP	Downward curling	25–40	2/5
C4-GFP	Curling of margin of leaves, small leaves	20–25	3/5
βC1-GFP	No apparent symptoms	No symptom after 30 days	0/5
CYVMV + CroYVMB	Leaf curl, marginal rolling, vein bending	9–10	5/5
pEarleyGate103 (Mock)	No symptoms	–	0/5

*Number of symptomatic plants/total number of plants. CroYVMB, croton yellow vein betasatellite; GFP, green fluorescent protein.

## Discussion

In the present investigation, CYVMV-encoded V2, C2, and C4 and its satellite CroYVMB-encoded βC1 have all exhibited properties as RNA silencing suppressors as evidenced by the restoration of the silenced GUS activity through a localized transient assay. Such transient agroinfiltration method using either GFP or GUS reporter is a standard protocol widely used for the functional analysis of silencing suppressors as well as pathogenic effectors (Voinnet et al., 1999; Reed et al., 2003; Korner et al., 2018). Begomovirus C2 and satellite βC1 homologs are nucleus-localized suppressors of both TGS and PTGS (Krake et al., 1998). We also found CYVMV C2 and CroYVMB βC1 proteins in the nucleus. They may suppress PTGS *via* cytosolic-related pathways. Inoculation of C2 with CYVMV caused chromosome condensation and a dilated nucleus. Similar observation was previously reported for tomato golden mosaic virus (TGMV) (Bass et al., 2000). Begomovirus V2 and C4 homologs have been reported to be cytoplasm-localized proteins that specifically suppress PTGS (Krake et al., 1998). We found that CYVMV V2 and C4 are localized in the cytoplasm and plasma membrane, respectively. They may suppress PTGS in different subcellular locations. It was reported that cotton leaf curl multan virus C4 can suppress both TGS and PTGS *via* interacting with SAM synthetase (Ismayil et al., 2018). Whether CYVMV C4 also acts as a dual-function silencing suppressor remains to be determined.

V2 is considered as the strongest PTGS suppressor encoded by begomoviruses (Luna et al., 2017). It may suppress PTGS by impairing the RDR6/SGS3 pathway (Luna et al., 2020). Although previous reports and our work all showed that the expression of V2 itself is sufficient to induce pathogenicity symptoms on host plants, this activity is independent of PTGS suppression function of V2 (Luna et al., 2020). There are also reports on certain begomoviruses that V2 can suppress both TGS and PTGS (Wang et al., 2018; Mubin et al., 2019). V2-mediated TGS suppression may be achieved by its physical interaction with host histone deacetylase 6 to suppress methylation (Wang et al., 2018; Yang et al., 2018).

V2 can suppress both local and systemic RNA silencing (Yang et al., 2018; Li et al., 2021), which is consistent with our observations that CYVMV V2 moves to the plasmodesmata in the presence of replicating virus and is required for its systemic movement within the host plants. However, there is also a report suggesting that the movement of tomato yellow leaf curl virus does not require its V2 protein (Hak et al., 2015).

Our work uncovers the novel feature of self-interaction and interaction with V1 of CYVMV V2. Together with its CYVMV-induced relocalization to the plasmodesmata, the DNA-protein and protein-protein interactions may promote the virus movement from the infected cell to a neighboring uninfected one (i.e., cell-to-cell movement). Since V1 functions as a CP, V2 possibly mediates the CYVMV movement *via* the direct interaction with V1. This model is consistent with our observation that the deletion of either V1 or V2 gene from CYVMV impairs virus mobility *in planta*. Since V1 and V2 gene transcription is controlled by a single viral promoter in the same direction (Ashraf et al., 2014; Li et al., 2018), their synchronized expression ensures *in vivo* interaction at the protein-protein level. V1/V2 single- or double-knockout constructs can replicate but cannot move systemically as evidenced by their amplification from inoculated area using a divergent primer set (abutting primers) designed from C1 ORF. For the mini viral replicon assay, this is a commonly used method (Singh et al., 2007; Kaliappan et al., 2012). The primers we used failed to amplify the parent plasmid due to its large size. The potential usage of the RCA technique might not be helpful as it would amplify both the parent plasmid as well as the episome. Restriction in viral movement was further evidenced through GFP tagged expression of both V1 and V2 deletion constructs. In the case of cotton leaf curl Kokhran virus, V2 and its CP (protein from V1 ORF) physically interact to facilitate the cell-to-cell movement of the virus (Poornima Priyadarshini et al., 2011). Our work thus demonstrates that V1 and V2 physically interact, and the absence of either protein hampers the cell-to-cell movement of the virus.

Pathogenicity determination assays showed that all three CYVMV-encoded silencing suppressors (C2, V2, and C4) are involved in the development of the host symptom, which is consistent with the previously reported pathogenesis roles of their orthologs from other begomoviruses. For example, the C2 protein of bhendi yellow vein mosaic virus plays an important role in symptom determination and viral genome replication in *N. benthamiana* plants (Chandran et al., 2014). A single amino acid mutation (i.e., S71A) in tomato yellow leaf curl virus V2 protein is sufficient to abolish its self-interaction, aggregation, and pathogenesis function (Zhao et al., 2018). The pathogenicity determination and PTGS suppression function of tomato leaf curl Guangdong virus C4 are regulated by its interaction with *N. benthamiana* receptor-like kinase BAM1 (Li et al., 2020). The symptom determination function of βC1 has been observed in other begomoviruses (Ding et al., 2009) but not for CYVMV in our investigation. A detailed study involving the molecular pathogenesis mechanism by each of CYVMV C2/V2/C4 proteins is still to be carried out.

In conclusion, through this study, we characterized four silencing suppressor proteins encoded by CYVMV and its cognate betasatellite, showed their subcellular localizations, and established their roles in pathogenicity. In addition, we demonstrated the self-interaction of V2 protein and the interaction between V2 and the CP (product of V1 ORF). The absence of either protein inhibited the cell-to-cell movement of the virus. In future studies, the identification of host factors interacting with these suppressor proteins and/or repressing the expression of their coding genes would deepen our understanding of CYVMV pathogenicity mechanisms and provide new clues for the development of disease management strategies.

## Data Availability Statement

The original contributions presented in the study are included in the article/Supplementary Material, further inquiries can be directed to the corresponding author/s.

## Author Contributions

HRP and AR conceived and designed the research. YZ, AR, HP, DM, and GK performed the experiments, collected the data, and analyzed the results. YZ, AR, HP, and HRP wrote the manuscript. BM and SM provided input to the experimental design and discussion. All the authors read and approved the final manuscript.

## Conflict of Interest

The authors declare that the research was conducted in the absence of any commercial or financial relationships that could be construed as a potential conflict of interest.

## Publisher’s Note

All claims expressed in this article are solely those of the authors and do not necessarily represent those of their affiliated organizations, or those of the publisher, the editors and the reviewers. Any product that may be evaluated in this article, or claim that may be made by its manufacturer, is not guaranteed or endorsed by the publisher.

## Supplementary Material

Supplementary Figure 1 Schematic strategy for croton yellow vein mosaic virus (CYVMV) V1 and V2 gene-knockout construct development. *Marked enzymes in multiple cloning sites (MCS) are also present in the virus genome portion so cannot be used for cloning.
