# Supplementary material for: Identification and Functional Analysis of Four RNA Silencing Suppressors in Begomovirus Croton Yellow Vein Mosaic Virus
Source: Front Plant Sci. 2022 Jan 7;12:768800. doi: 10.3389/fpls.2021.768800 (PMC8777275; doi:10.3389/fpls.2021.768800)
Supplement: Supplementary Figure 1 — Schematic strategy for croton yellow vein mosaic virus (CYVMV) V1 and V2 gene-knockout construct development. *Marked enzymes in multiple cloning sites (MCS) are also present in the virus genome portion so cannot be used for cloning. [file Image_1.PDF]

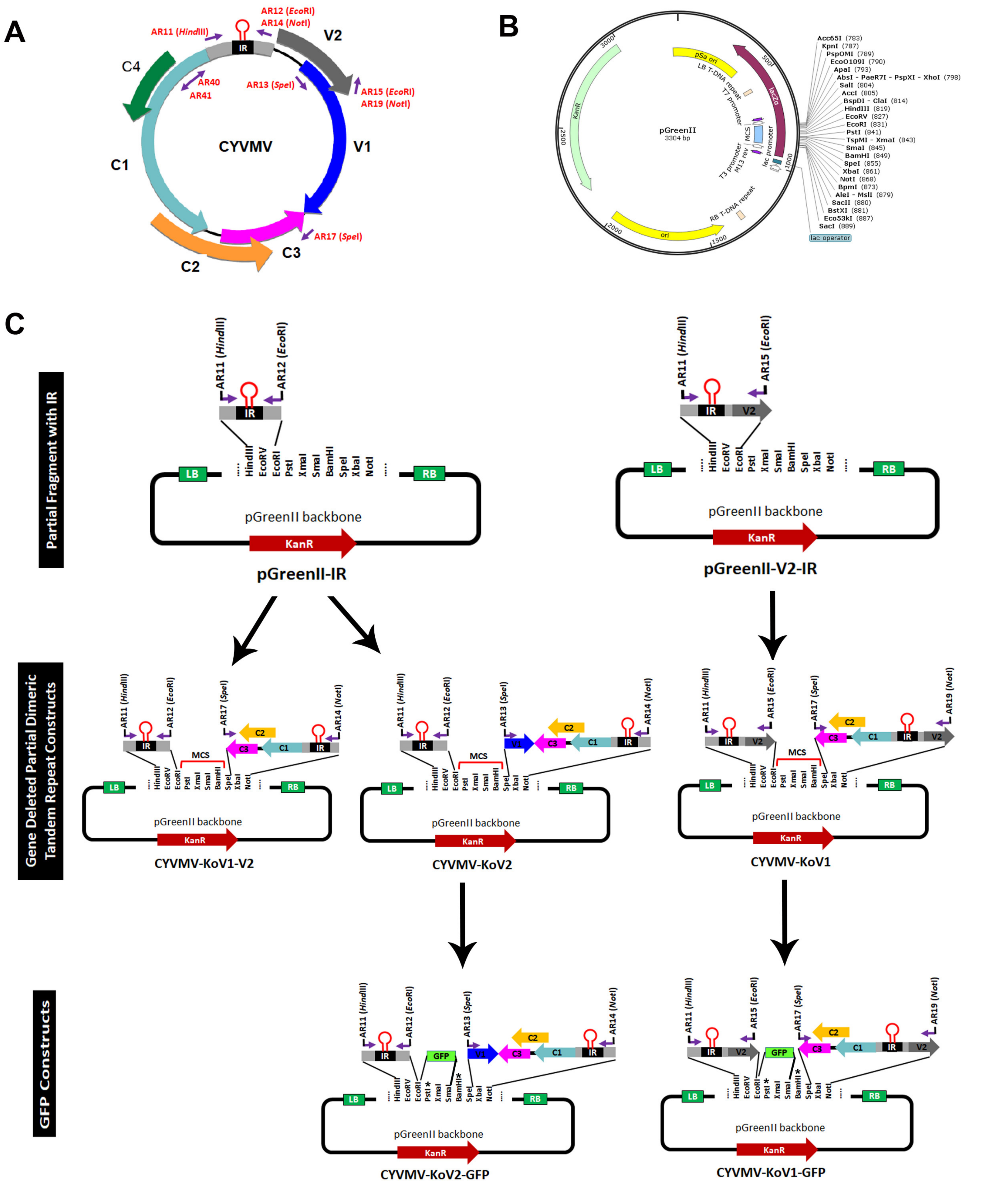

A
AR11 (HindIII)
AR12 (EcoRI)
AR14 (NotI)
IR
V2
C4
AR40
AR41
AR13 (SpeI)
AR15 (EcoRI)
AR19 (NotI)
CYVMV
V1
C1
AR17 (SpeI)
C2
C3
B
pGreenII
3304 bp
KanR
pSa ori
LB T-DNA repeat
T7 promoter
MCS
lacZα
lac promoter
T3 promoter
M13 rev
RB T-DNA repeat
ori
lac operator
Acc65I (783)
KpnI (787)
PspOMI (789)
EcoO109I (790)
ApaI (793)
AbsI - PaeR7I - PspXI - XhoI (798)
SalI (804)
AccI (805)
BspDI - ClaI (814)
HindIII (819)
EcoRV (827)
EcoRI (831)
PstI (841)
TspMI - XmaI (843)
SmaI (845)
BamHI (849)
SpeI (855)
XbaI (861)
NotI (868)
BpmI (873)
AleI - MslI (879)
SacII (880)
BstXI (881)
Eco53kI (887)
SacI (889)
C
Partial Fragment with IR
pGreenII backbone
KanR
LB
RB
pGreenII-IR
pGreenII-V2-IR
Gene Deleted Partial Dimeric Tandem Repeat Constructs
MCS
CYVMV-KoV1-V2
CYVMV-KoV2
CYVMV-KoV1
GFP Constructs
GFP
CYVMV-KoV2-GFP
CYVMV-KoV1-GFP
