# Supplementary material for: Identification and Functional Analysis of Four RNA Silencing Suppressors in Begomovirus Croton Yellow Vein Mosaic Virus
Source: Front Plant Sci. 2022 Jan 7;12:768800. doi: 10.3389/fpls.2021.768800 (PMC8777275; doi:10.3389/fpls.2021.768800)
Supplement: Supplementary file 2 [file Table_1.DOCX]

**Supplementary Table 1 |** PCR Primers used in this study.

|  | Primer sequence (5’-3’) | Expected amplicon size (bp**)** |
| --- | --- | --- |
| V2-For | CACCATGTGGGATCCATTAGT | 357 |
| V2-Rev | GGAACATCTGGGCTTCTGTAC |  |
| C2-For | CACCATGCGACCTTTATCAC | 405 |
| C2-Rev | AATACCCTTAAGAAATGACCAGTC |  |
| C4-For | CACCATGGGTCTCTGCATATCC | 258 |
| C4-Rev | GGGCCTCTGCTGCTGCATC |  |
| βC1-For | CACCATGACGATCATATATCAG | 357 |
| βC1-Rev | CATATTTACACATTTATACACAACATATTC |  |
|  |  |  |
| AR11 | AAAAGCTTATTGAATTGGGGACACTC |  |
| AR12 | AAGAATTCTGTGCGCAATACACTACTT |  |
| AR13 | AAACTAGTATGTCGAAGCGTCCAGCAG |  |
| AR14 | AAGCGGCCGCTGTGCGCAATACACTACTT |  |
| AR15 | AAGAATTCTTAGGGACATCTGGGCTTC |  |
| AR17 | AAACTAGTTTAATAAAGATTGAATTTTATTGAATATG |  |
| AR19 | AAGCGGCCGCTTAGGGACATCTGGGCTTC |  |
| AR20 | AAGAATTCCATGGAGTCAAAGATTCAAATAG |  |
| AR21 | AAGGATCCCCCGATCTAGTAACATAGATGAC |  |
| AR40 | GCCTTCGAGGGTGATGAAGG |  |
| AR41 | CCACTCTACTCAGGTTCC |  |
